# Real-life impact of highly effective CFTR modulator therapy in children with cystic fibrosis

**DOI:** 10.3389/fphar.2023.1176815

**Published:** 2023-05-09

**Authors:** Margarete Olivier, Alexandra Kavvalou, Matthias Welsner, Raphael Hirtz, Svenja Straßburg, Sivagurunathan Sutharsan, Florian Stehling, Mathis Steindor

**Affiliations:** ^1^ Pediatric Pulmonology and Sleep Medicine, Cystic Fibrosis Center, Children’s Hospital, University of Duisburg-Essen, Essen, Germany; ^2^ Department of Pulmonary Medicine, Adult Cystic Fibrosis Center, University Hospital Essen—Ruhrlandklinik, University of Duisburg-Essen, Essen, Germany; ^3^ Pediatric Endocrinology, Children’s Hospital, University of Duisburg-Essen, Essen, Germany

**Keywords:** tio real-life, modulator, children, ivacaftor, tezacaftor, elexacaftor, cystic fibrosis

## Abstract

**Introduction:** Recently, cystic fibrosis transmembrane regulator modulator therapy with elexacaftor/tezacaftor/ivacaftor has become available for children with cystic fibrosis (CF) carrying at least one *F508del* mutation.

**Objective:** To assess the intermediate term effects of elexacaftor/tezacaftor/ivacaftor in children with cystic fibrosis in a real-world setting.

**Methods:** We performed a retrospective analysis of records of children with cystic fibrosis, who started elexacaftor/tezacaftor/ivacaftor between 8/2020 and 10/2022. Pulmonary function tests, nutritional status, sweat chloride and laboratory data were assessed before, 3 and 6 months after the start of elexacaftor/tezacaftor/ivacaftor respectively.

**Results:** Elexacaftor/tezacaftor/ivacaftor was started in 22 children 6–11 years and in 24 children 12–17 years. Twenty-seven (59%) patients were homozygous for *F508del* (F/F) and 23 (50%) patients were transitioned from ivacaftor/lumacaftor (IVA/LUM) or tezacaftor/ivacaftor (TEZ/IVA) to elexacaftor/tezacaftor/ivacaftor. Overall, mean sweat chloride concentration decreased by 59.3 mmol/L (95% confidence interval: −65.0 to −53.7 mmol/L, *p* < 0.0001) under elexacaftor/tezacaftor/ivacaftor. Sweat chloride concentration also decreased significantly after transition from IVA/LUM or TEZ/IVA to elexacaftor/tezacaftor/ivacaftor (−47.8 mmol/l; 95% confidence interval: −57.6 to −37.8 mmol/l, *n* = 14, *p* < 0.0001). Sweat chloride reduction was more marked in children with the F/F than in those with the F/MF genotype (69.4 vs 45.9 mmol/L, *p* < 0.0001). At 3 months follow-up, body-mass-index-z-score increased by 0.31 (95% CI, 0.2–0.42, *p* < 0.0001) with no further increase at 6 months. BMI-for-age-z-score was more markedly improved in the older group. Overall pulmonary function (percent predicted FEV_1_) at 3 months follow-up increased by 11.4% (95% CI: 8.0–14.9, *p* < 0.0001) with no further significant change after 6 months. No significant differences were noted between the age groups. Children with the F/MF genotype had a greater benefit regarding nutritional status and pulmonary function tests than those with the F/F genotype. Adverse events led to elexacaftor/tezacaftor/ivacaftor dose reduction in three cases and a temporary interruption of therapy in four cases.

**Conclusion:** In a real-world setting, elexacaftor/tezacaftor/ivacaftor therapy had beneficial clinical effects and a good safety profile in eligible children with cystic fibrosis comparable to previously published data from controlled clinical trials. The positive impact on pulmonary function tests and nutritional status seen after 3 months of elexacaftor/tezacaftor/ivacaftor therapy was sustained at 6 months follow-up.

## Introduction

Cystic fibrosis (CF) is an autosomal recessive multi-system disease, which results from mutations in the CF transmembrane conductance regulator (*CFTR*) gene (Riordan et al., 1989). While 300 CF-causing mutations and >2,000 CFTR mutations are known, the *F508del*-CFTR mutation is by far the most frequent being present in nearly 90% of people with CF (pwCF) (Riordan, 2008). Patients with *F508del-CFTR* mutations have decreased quantity and function of the CFTR protein (Ratjen et al., 2015) leading to severe disease manifestations, e.g., inborn exocrine pancreatic insufficiency, growth impairment, and progressive lung disease (Ratjen et al., 2015). Although substantial progress in the symptomatic care of pwCF was achieved over the last decades (MacKenzie et al., 2014) high disease burden and reduced life expectancy in these patients underlined the need for targeted CFTR therapies (Rang et al., 2020).

This goal was first met with the introduction of the CFTR-potentiator ivacaftor (IVA), which efficiently enhanced CFTR channel gating in the small group of CF patients with CFTR gating mutations (Ramsey et al., 2011) and achieved substantial improvements in nutritional status and pulmonary function (De Boeck et al., 2014; McKone et al., 2014). Subsequently, CFTR correctors, such as lumacaftor (LUM) and tezacaftor (TEZ) were developed, which improved CFTR processing and trafficking to epithelial surfaces. In dual combinations with ivacaftor, these substances were moderately effective in patients homozygous for *F508del* (F/F) (LUM/IVA) and in patients carrying a residual function mutation (TEZ/IVA) (Wainwright et al., 2015; Rowe et al., 2017). Since 2019, the triple-substance regimen of TEZ/IVA and the next-generation corrector elexacaftor (ELX) has been proven safe and effective (Heijerman et al., 2019; Middleton et al., 2019; Sutharsan et al., 2022) in adolescents and adults with the F/F genotype as well as in patients who were heterozygous for *F508del* and a minimal function (MF) *CFTR* mutation*.* ELX/TEZ/IVA (ETI) treatment resulted in so far unprecedented improvements in pulmonary function tests, respiratory symptoms and CFTR function reflected by the sweat chloride concentration, giving a new perspective to pwCF carrying at least one *F508del* mutation (Middleton et al., 2019; Sutharsan et al., 2022). However, in view of the very early onset of CF organ disease it was evident that ETI therapy should be offered to younger children and ultimately infants to tackle and prevent the sequelae of CFTR dysfunction (VanDevanter et al., 2016).

In an open-label phase 3 study, the safety, pharmacokinetics, and efficacy of ETI was examined in children aged 6 through 11 years with either F/MF or F/F genotypes (Zemanick et al., 2021). ETI therapy was safe and led to significant improvements in pulmonary function tests, sweat chloride concentration, lung clearance index (LCI) and nutritional status. In this trial, the therapeutic effects were comparable to those seen in adult patients (Zemanick et al., 2021). In a subsequent randomized, placebo-controlled trial including children 6–11 years with F/MF genotypes, the positive effects on pulmonary function, LCI, respiratory symptoms and sweat chloride were confirmed (Mall et al., 2022). Again, no safety concerns arose in the course of the trial compared to children receiving standard CF care. Consequently, in January 2022 the EMA approved the use of ETI for treatment of children with CF from the age of six. In line with most pediatric CF-centers, we intended to initiate ETI immediately in our children eligible for this treatment. In the present study, we report our experience in children and adolescents with CF during the first 6 months of ETI therapy. In contrast to the previously described controlled trials, our analysis investigates the post-approval efficacy of ETI across a heterogeneous collective of young CF-patients in a real-life setting.

## Methods

We retrospectively investigated the records of all children with CF of our Cystic Fibrosis Centre for the period August 2020 to January 2023. All patients fulfilled the inclusion criteria, which were established diagnosis of CF (sweat chloride concentration ≥60 mmol/L and 2 CF-defining mutations), proof of at least one *F508del* mutation, and age ≥6 years, documenting eligibility for ETI treatment. Children participating in a clinical trial were excluded. ETI dosing was performed according to official dosage recommendations: children weighing <30 kg received ELX 100 mg once daily, TEZ 50 mg once daily, and IVA 75 mg every 12 h, whereas children weighing ≥30 kg received the full adult daily dose (ELX 200 mg once daily, TEZ 100 mg once daily, and IVA 150 mg every 12 h). Data for biometry, percentage of predicted (pp) FEV_1_, alanine aminotransferase (ALT) or aspartate aminotransferase (AST), alkaline phosphatase (AP), creatinkinase (CK) and concomitant nebulized medication were collected before the start of ETI and at 3 and 6 months follow-up (F/U 1 and F/U 2) after initiation of ETI therapy. The results of hepatic sonography before start of ETI were reviewed. Sweat chloride concentrations in a modulator-naïve state were compared to results after onset of ETI therapy. Reports of adverse effects during treatment with ETI were extracted from patient records. First, data were analyzed across the entire cohort. Subsequently, data from patients aged six through 11 years were compared to data from patients ≥12 years. Also, data of children with the F/F genotype were compared to *F508del*-heterozygous patients. Finally, the change in ppFEV_1_ at 3 and 6 months F/U was analyzed according to baseline ppFEV1 (ppFEV_1_ < 80% and ≥80%). This study was approved by the local ethics committee of the University of Duisburg‐Essen (Study‐No. 23-11141‐BO).

### Statistical analysis

GraphPad Prism Version 7 (GraphPad Software Inc., Boston, US) was used to analyze and visualize the data. Paired and independent *t*-tests, respectively, were used to analyze the statistical significance of the study parameters. A *p* < 0.05 was considered statistically significant. Pearson correlation coefficients were calculated using Excel 2016 1.0. All analyses were corrected for multiple testing controlling the two-sided false discovery rate (FDR) at *p* < 0.05.

## Results

### Entire cohort (6–17 years)

**FIGURE 1:**
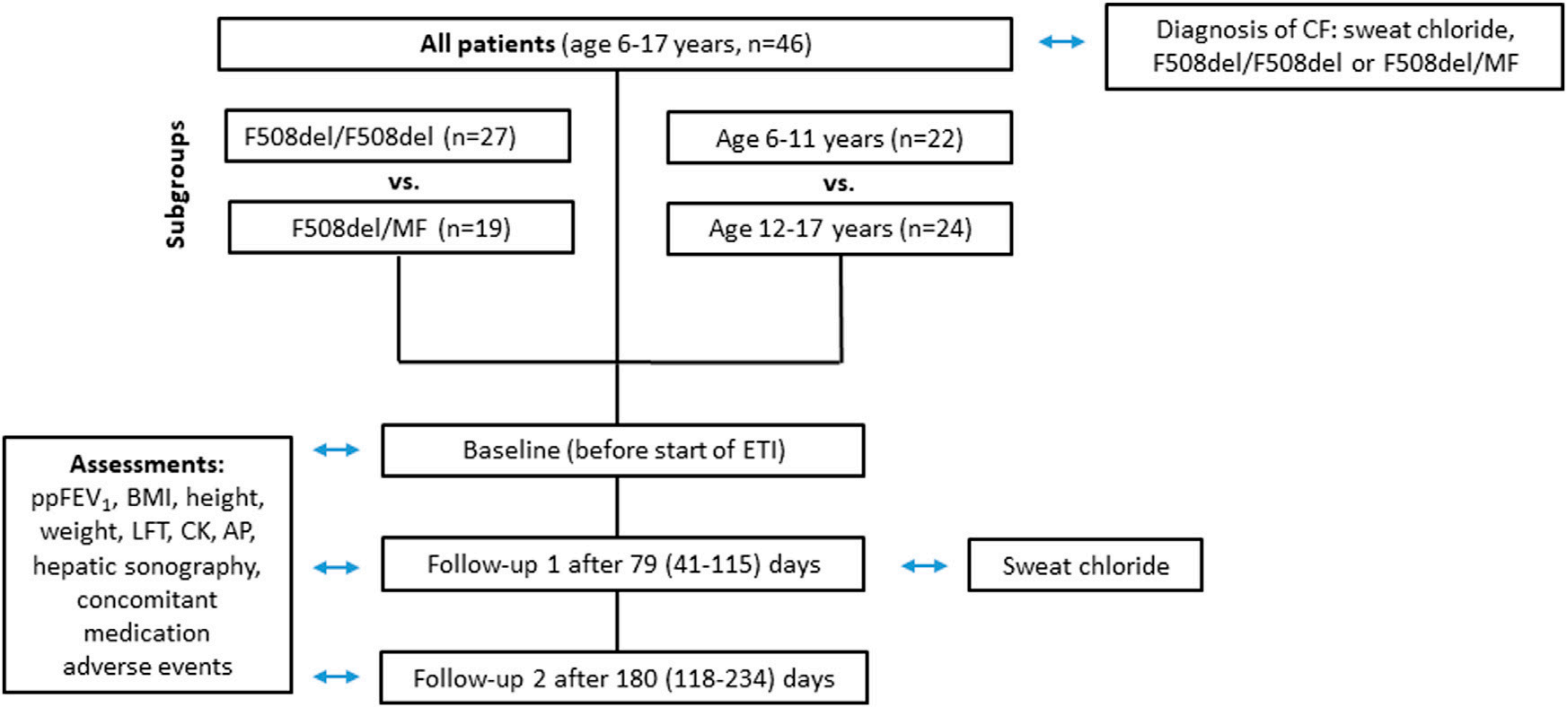
Flowchart of parameters assessed at baseline, follow-up 1 and follow-up 2 for all patients groups.

Forty-six patients (19 male, 27 female) were included in the data analysis (Figure 1). Eight patients were excluded due to current clinical trial involvement. Patient characteristics and concomitant medications are given in Table 1 and Table 2 respectively. Mean age of patients starting ETI after the approval for the age group ≥12 years in 2020 was 14.3 years (range 12.1—17.1) while mean age of patients starting ETI after the approval for children ≥6 years in 2022 was 8.6 years (range 6.0–11.9) years. Twenty-seven patients were homozygous for *F508del* (F/F). Prior modulator use was LUM/IVA in 21 patients and TEZ/IVA in two patients. The two follow-up examinations (F/U 1 and F/U 2) occurred after a mean of 79 days (range 41–115) and 180 days (range 118–234), respectively, following start of ETI therapy.

**TABLE 1:** Baseline characteristics.

Characteristcs	All patients	6–11 years	12–17 years
number	46	22	24
female/male	27/19	14/8	13/11
Age (years) at baseline	11.5 (6.0–17.1)	8.6 (6.0–11.9)	14.3 (12.1–17.1)
F/U 1 (days)	79 (41–115)	88 (38–112)	80 (41–108)
F/U 2 (days)	180 (118–234)	191 (150–210)	175 (118–234)
Genotype			
F/F	27	16	11
F/MF	19	6	13
Prior modulator use			
LUM/IVA	21	15	6
TEZ/IVA	2		2

F/F, Patients homozygous for F508del; F/MF, Patients heterozygous for F508del and a minimal function mutation; LUM, Lumacaftor; IVA, ivacaftor.

**TABLE 2:** Concomitant medication.

Concomitant medication	—	Visit reported	Use (%)
Hypertonic saline			
6–11 years (*n* = 22)	Twice/once daily	Baseline	19/3 (100)
		F/U 1	20/2(100)
		F/U 2	20/2(100)
12–17 years (*n* = 24)	Twice/once daily	Baseline	21/3 (100)
		F/U 1	21/3(100)
		F/U 2	23/1 (100)
Dornase alfa			
6–11 years	—	Baseline	13 (59)
	—	F/U 1	13 (59)
	—	F/U 2	13 (59)
12–17 years	—	Baseline	20 (83)
	—	F/U 1	18 (83)
	—	F/U 2	18 (83)
Inhaled antibiotics			
6–11 years	—	Baseline	5(23)
	—	F/U 1	4 (18)
	—	F/U 2	5 (23)
12–17 years	—	Baseline	6 (25)
	—	F/U 1	4 (17)
	—	F/U 2	3 (13)

F/U 1, Follow-up visit after 3 months of ETI treatment; F/U 2, Follow-up visit after 6 months of ETI treatment.

**FIGURE 2:**
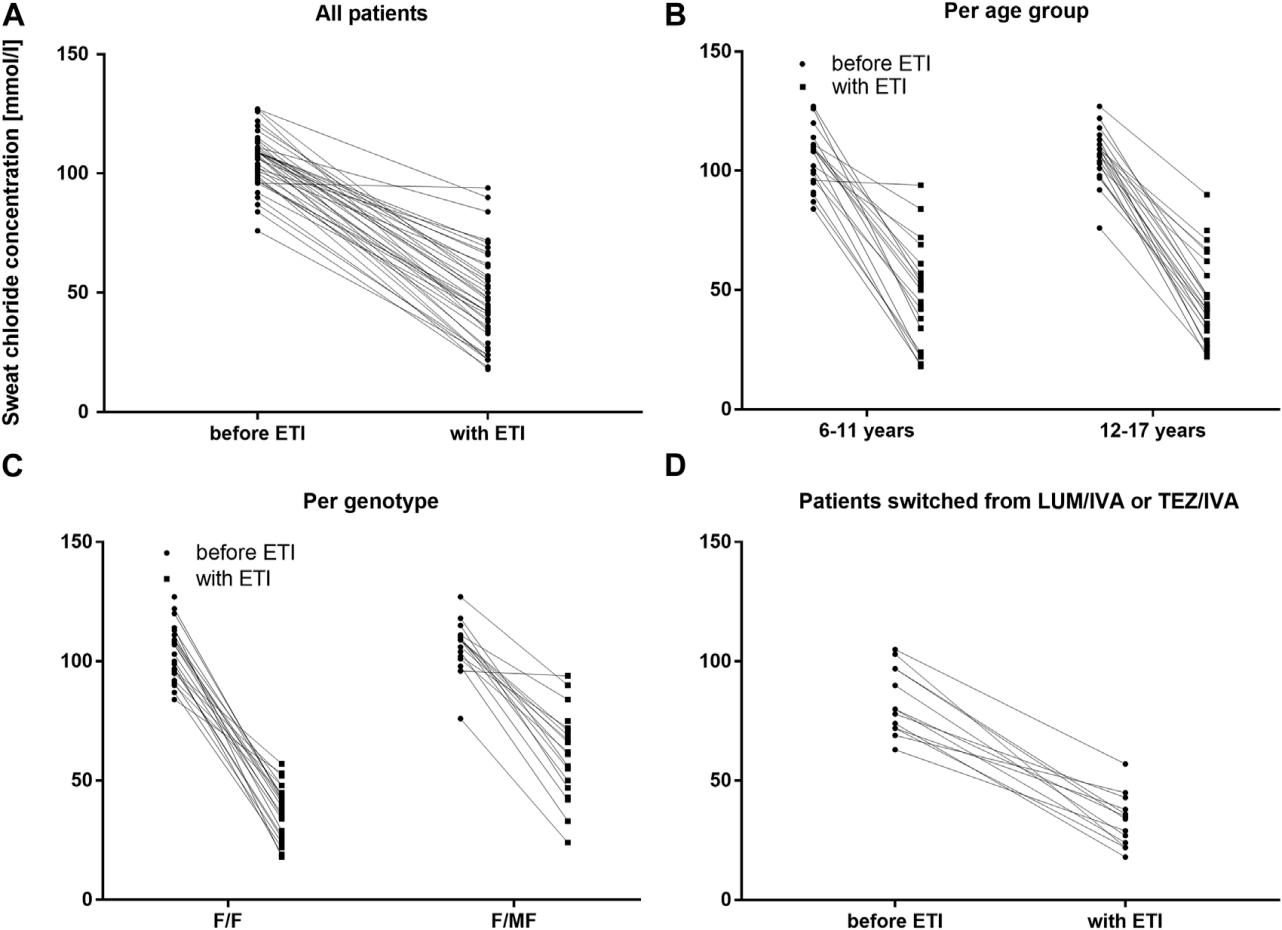
Sweat chloride concentrations before and with ETI therapy in mmol/L **(A)** Across the entire cohort before ETI (circles) and with ETI (squares) **(B)** In patients 6–11 years and in patients 12–17 years at baseline before ETI (circles) and with ETI (squares) **(C)** In patients with the F/F genotype and in patients with F/MF genotypes before ETI (circles) and with ETI (squares) **(D)** In patients switched from LUM/IVA or TEZ/IVA before ETI (circles) and with ETI (squares).

Two extreme outliers concerning the baseline sweat chloride concentration (170 mmol/l and 220 mmol/l) were excluded from further analysis. At baseline, no children had sweat chloride concentrations below the diagnostic threshold for CF of 60 mmol/L, even if pretreated with LUM/IVA or TEZ/IVA. Across the entire cohort, mean sweat chloride concentration decreased by 59.3 mmol/L (95% confidence interval [CI]: −65.0 to −53.7. mmol/L, *n* = 42) (*p* < 0.0001) compared to the modulator-naïve state (Table 3; Figure 2A). In children, who were switched from LUM/IVA or TEZ/IVA to ETI sweat chloride concentration also decreased significantly by 47.8 mmol/l (95% CI: −57.6 to −37.8 mmol/l, *n* = 14, *p* < 0.0001) (Table 3; Figure 2D). At F/U 1 and F/U 2, there was no significant correlation between the decrease in sweat chloride concentrations and the change in ppFEV1 (*r* = 0.08 and *r* = 0.15), weight (*r* = 0.23 and *r* = 0.28), weight for age z-score (*r* = 0.31 and *r* = 0.27), BMI (*r* = 0.02 and *r* = 0.05) or BMI for age-z-score (*r* = − 0.06 and *r* = 0.01) when considering a correction for multiple testing. Sweat test at follow-up was borderline in 23 patients, above 60 mmol/l in 12 patients, and normal in 10 patients. In one patient, follow-up sweat sampling failed due to insufficient sweat quantity despite several attempts.

**TABLE 3:** Patient characteristics at baseline, follow-up 1 and follow–up 2.

Parameter	Patient categories	Baseline (mean, SD)	F/U 1 (mean, SD)	Change from BL at F/U 1 [mean (95% CI)]	*p*-value	F/U 2 (mean, SD)	Change from BL at F/U2 [mean (95% CI)]	*p*-value	*p*-value F/U1 vs F/U 2
ppFEV1	all patients	81.4 (15.6)	92.8 (15.5)	11.4 (8.0—14.9) (*n* = 45)	*p* < 0.0001	93.7 (14.46)	12.8 (9.1—16.5) (*n* = 41)	*p* < 0.0001	*p* = 0.2629
[%]	6–11 years	85.0 (16.2)	94.2 (16.3)	9.8 (4.6—15.1) (*n* = 22)	*p* = 0.0009	96.8 (14.29)	12.9 (7.1—18.7) (*n* = 18)	*p* = 0.0002	*p* = 0.1075
	12–17 years	77.6 (14.2)	91.3 (14.7)	13.0 (8.1—17.8) (*n* = 23)	*p* < 0.0001	91.1 (14.4)	12.8 (7.6—18.0) (*n* = 23)	*p* < 0.0001	*p* = 0.8912
	F/F	83.4 (15.5)	91.6 (15.6)	8.4 (4.8–12.1) (*n* = 26)	*p* < 0.0001	92.0 (14.6)	9.3 (5.1–13.5) (*n* = 23)	*p* = 0.0001	*p* = 0.4130
	F/MF	78.5 (15.6)	94.1 (15.7)	15.5 (9.1–22.0) (*n* = 19)	*p* < 0.0001	95.9 (14.5)	17.3 (11.0–23.7) (*n* = 18)	*p* < 0.0001	*p* = 0.4634
	ppFEV1 < 80%	65.8 (10.3)	81.3 (11.6)	15.6 (10.3–20.8) (*n* = 18)	*p* < 0.0001	83.9 (11.4)	18.9 (13.5-24.3 (*n* = 17)	*p* < 0.0001	*p* = 0.0541
	ppFEV 1 ≥ 80%	91.7 (7.7)	100.4 (13.0)	8.7 (4.1–13.2) (*n* = 27)	*p* < 0.0006	100.7 (12.4)	8.5 (4.0–13.1) (*n* = 24)	*p* < 0.0007	*p* = 0.9566
weight-for-age	all patients	−0.56 (0.98)	−0.32 (0.91)	0.24 (0.15–0.33) (*n* = 46)	*p* < 0.0001	−0.18 (0.95)	0.32 (0.18–0.47) (*n* = 42)	*p* < 0.0001	*p* = 0.1461
[z-Score]	6–11 years	−0.40 (0.90)	−0.26 (0.84)	0.14 (0.01–0.27) (*n* = 22)	*p* = 0.0365*	−0.07 (0.81)	0.18 (−0.01–0.37) (*n* = 18)	*p* = 0.0657	*p* = 0.8015
	12–17 years	−0.72 (1.05)	−0.37 (0.99)	0.34 (0.22—0.46) (*n* = 24)	*p* < 0.0001	−0.27 (1.06)	0.43 (0.22–0.64) (*n* = 24)	*p* = 0.0003	*p* = 0.1243
	F/F	−0.46 (0.93)	−0.33 (0.92)	0.13 (0.06—0.21) (*n* = 27)	*p* = 0.0013	−0.21 (0.89)	0.13 (−0.01–0.27) (*n* = 24)	*p* = 0.0610	*p* = 0.7524
	F/MF	−0.69 (1.05)	−0.29 (0.92)	0.40 (0.23–0.57) (*n* = 19)	*p* = 0.0001	−0.14 (1.05)	0.58 (0.33–0.83) (*n* = 18)	*p* = 0.0002	*p* = 0.0311*
BMI-for-age	all patients	−0.15 (0.84)	0.16 (0.72)	0.31 (0.20—0.42) (*n* = 46)	*p* < 0.0001	0.18 (0.88)	0.38 (0.19–0.56) (*n* = 42)	*p* = 0.0002	*p* = 0.4719
[z-score]	6–11 years	0.08 (0.70)	0.28 (0.57)	0.20 (0.04—0.36) (*n* = 22)	*p* = 0.0141	0.23 (0.78)	0.19 (−0.04–0.43) (*n* = 18)	*p* = 0.1035	*p* = 0.5677
	12–17 years	−0.37 (0.91)	0.04 (0.82)	0.41 (0.27—0.55) (*n* = 24)	*p* < 0.0001	0.15 (0.97)	0.51 (0.24–0.79) (*n* = 24)	*p* = 0.0007	*p* = 0.2202
	F/F	−0.04 (0.82)	0.13 (0.82)	0.17 (0.09–0.24) (*n* = 27)	*p* < 0.0001	0.05 (0.96)	0.12 (−0.03–0.27) (*n* = 24)	*p* = 0.1113	*p* = 0.3045
	F/MF	−0.32 (0.85)	0.20 (0.56)	0.51 (0.30–0.72) (*n* = 19)	*p* < 0.0001	0.37 (0.77)	0.72 (0.38–1.06) (*n* = 18)	*p* = 0.0003	*p* = 0.0837
height-for-age	all patients	−0.75 (1.18)	−0.75 (1.14)	0 (−0.06–0.06) (*n* = 46)	*p* = 0.9007	−0.60 (1.03)	0.01 (−0.06–0.08) (*n* = 42)	*p* = 0.7544	*p* = 0.7252
[z-score]	6–11 years	−0.78 (1.11)	−0.82 (1.09)	−0.04 (−0.11-0.04) (*n* = 22)	*p* = 0.3254	- 0.43 (0.80)	0.03 (−0.05–0.12) (*n* = 18)	*p* = 0.3910	*p* = 0.0061
	12–17 years	−0.72 (1.26)	−0.69 (1.21)	0.03 (−0.07–0.12) (*n* = 24)	*p* = 0.5615	- 0.72 (1.18)	−0.01 (−0.12-0.10) (*n* = 24)	*p* = 0.8933	*p* = 0.5359
	F/F	−0.74 (1.20)	−0.77 (1.15)	−0.03 (−0.11-0.06) (*n* = 27)	*p* = 0.5136	−0.49 (0.91)	0.01 (−0.10–0.12) (*n* = 24)	*p* = 0.8611	*p* = 0.2256
	F/MF	−0.76 (1.19)	−0.73 (1.17)	0.03 (−0.06–0.12) (*n* = 19)	*p* = 0.5061	−0.74 (1.19)	0.01 (−0.07–0.10) (*n* = 18)	*p* = 0.7583	*p* = 0.7489
Sweat chloride	all patients	105.8 (11.2.)	46.5 (19.2)	−59.3 (−65.0 to −53.7) (*n* = 42)	*p* < 0.0001	—	—	—	—
[mmol/l]	6–11 years	105.7 (11.5)	47.0 (20.8)	−58.7 (−67.8 to −49.5) (n = 22)	*p* < 0.0001	—	—	—	—
	12–17 years	106.0 (11.2)	45.9 (17.9)	−60.1 (−67.3 to −52.8) (*n =* 20)	*p* < 0.0001	—	—	—	—
	F/F	105.8 (11.8)	36.4 (11.7)	−69.4 (−74.3 to −64.5) (*n* = 24)	*p* < 0.0001	—	—	—	—
	F/MF	105.9 10.7)	59.9(19.3)	−45.9 (−54.2 to −37.7) (*n* = 18)	*p* < 0.0001	—	—	—	—
	Modulator switch	81.7 (14.7)	33.9 (11.9)	−47.8 (−57.6 to −37.8) (*n* = 14)	*p* < 0.0001	—	—	—	—

BMI, body mass index; F/F, Patients homozygous for F508del; F/MF, Patients heterozygous for F508del and a minimal function mutation; ppFEV1, percent predicted FEV1; SD, standard deviation; F/U 1, Follow-up visit after 3 months of ETI treatment; F/U 2, Follow-up visit after 6 months of ETI treatment; *, not statistically significant after correction for multiple tests.

**FIGURE 3:**
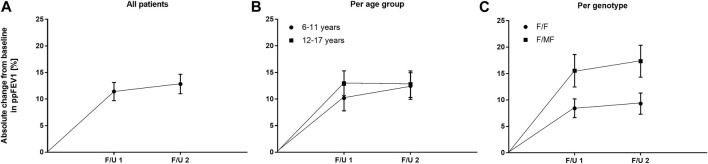
Absolute change in mean ppFEV1 before start of ETI and at F/U 1 and F/U 2 **(A)** Across the entire cohort **(B)** In patients 6–11 years (circles) and in patients 12–17 years (squares) at baseline **(C)** In patients with the F/F genotype (circles) and in patients with F/MF genotypes (squares).

With regard to pulmonary function, ETI therapy led to a significant improvement in ppFEV_1_ compared to baseline. At F/U 1, ppFEV_1_ increased by a mean of 11.4 percentage points (95% CI: 8.0–14.9, *n* = 45, *p* < 0.0001). This effect was sustained at 6-month-follow-up with a mean increase in ppFEV_1_ of 12.8% (95% CI: 9.1–16.5, *n* = 41, *p* < 0.0001) compared to baseline. There was no significant difference between the 2 F/U visits regarding pulmonary function tests (Table 3; Figure 3A). Eighteen patients had ppFEV_1_ < 80% at baseline whereas ppFEV_1_ was ≥80% in 28 patients. Both groups experienced a significant increase in ppFEV_1_ at both follow-up examinations after start of ETI. At F/U 1, the increase in ppFEV_1_ in patients with lower baseline pulmonary function was 6.9% higher than in the group with higher baseline ppFEV_1_ (15.6% *versus* 8.7%, 95% CI: 0.06–13.7, *p* < 0.048, n. s after correction for multiple testing). At 6 months F/U, there was a significantly higher increase in ppFEV_1_ in children with baseline pulmonary function <80% compared to those with higher baseline pulmonary function (18.9% *versus* 8.5%, 95% CI 3.5–17.2, *p* < 0.004). No further increase occurred in these subgroups between F/U 1 and F/U 2.

**FIGURE 4:**
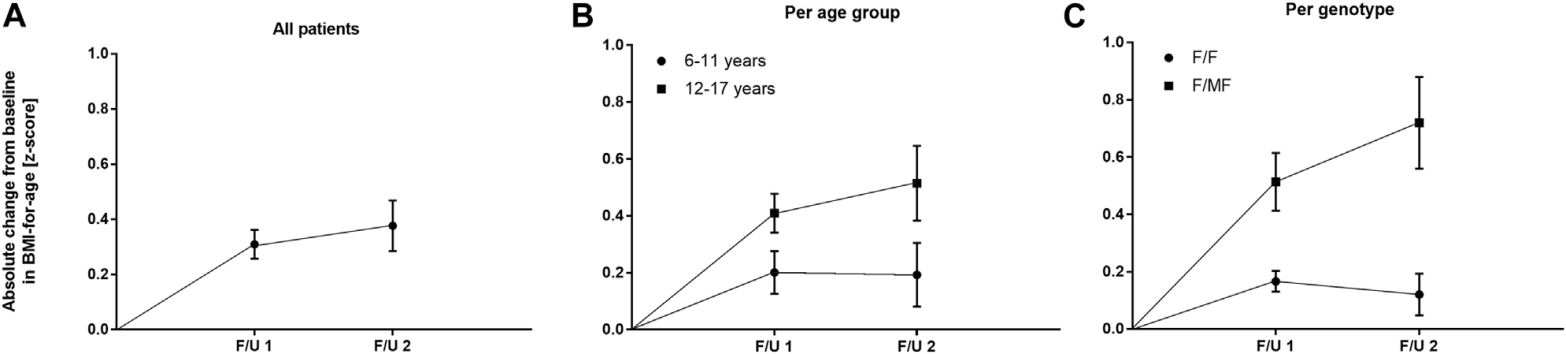
Absolute change in BMI-for-age z-score before start of ETI and at F/U 1 and F/U 2 **(A)** Across the entire cohort **(B)** In patients 6–11 years (circles) and in patients 12–17 years (squares) at baseline **(C)** In patients with the F/F genotype (circles) and in patients with F/MF genotypes (squares).

**FIGURE 5:**
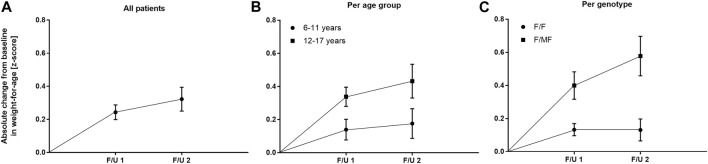
Absolute change in weight-for-age z-score before start of ETI and at F/U 1 and F/U 2 **(A)** Across the entire cohort **(B)** In patients 6–11 years (circles) and in patients 12–17 years (squares) at baseline **(C)** In patients with the F/F genotype (circles) and in patients with F/MF genotypes (squares).

Significant improvements were seen in BMI-for-age and weight-for-age *z*-scores at F/U 1, reaching a plateau through F/U 2. Height-for-age *z*-scores remained unchanged at F/U 1 (*p* = 0.901) and F/U 2 (*p* = 0.754). Specifically, ETI resulted in a BMI *z*-score that was 0.31 higher in comparison to baseline at F/U 1 (95% CI: 0.2–0.42, *n* = 46, *p* < 0.0001). Through F/U 2, BMI-for-age *z*-score was maintained without further significant increase (mean difference to baseline 0.38, 95% CI: 0.19 to 0.56, *n* = 42, *p* < 0.001) (Table 3; Figure 4A). Similar to BMI, sustained improvement in weight-for-age *z*-score was seen through F/U 2, with a mean difference of 0.32 relative to baseline (95% CI: 0.18–0.47, *n* = 42, *p* < 0.0001). The interim analysis at F/U 1 revealed a mean increase of 0.24 compared to baseline (95% CI: 0.15 to 0.33, *n* = 46, *p* < 0.0001). Again, no marked differences between 3 and 6 months were noticed (Table 3, Figure 5A).

With regard to concomitant medication there was no relevant change in the use of hypertonic saline, dornase alfa and nebulized antibiotics after the onset of ETI therapy.

### Subgroup analyses

#### Age groups (6–11 years versus ≥ 12 years)

There was no significant difference between the age groups regarding the effect of ETI on sweat chloride concentration at F/U 1, with a mean decrease of 58.7 mmol/l (95% CI −67.8–−49.5; *n* = 22, *p* < 0.0001) in the patients 6—11 years and a mean decrease of 60.1 mmol/l (95% CI—67.3–−52.8, *n* = 20, *p* < 0.0001) in the elder age group (*p* = 0.21). (Table 3; Figure 2B). In addition, the age groups did not differ significantly with regard to changes in pulmonary function tests as assessed by ppFEV_1_ at F/U 1 (*p* = 0.37) and F/U 2 (*p* = 0.98). In children 6—11 years ppFEV_1_ improved by 9.8% (95% CI 4.6—15.1; *n* = 22, *p* < 0.001) at F/U 1, whereas the elder children experienced an increase in ppFEV_1_ of 13.0% (95% CI 8.1—17.8, *n* = 20; *p* < 0,0001 (Table 3; Figure 3B).

In the younger group of patients, there was a trend toward an increase in weight-for-age *z*-scores at F/U 1, with a mean difference of 0.14 (*p* = 0.0365, n. s after correction for multiple testing). This increase was sustained at F/U 2 although again not reaching statistical significance (*p* = 0.0657) (Table 3; Figure 5B). With respect to BMI-for-age *z*-score, there was a mean difference of 0.2 above the baseline (95% CI: 0.04–0.36, *n* = 22, *p* = 0.0141), whereas the absolute change from baseline at F/U 2 was not statistically significant (Table 3; Figure 4B).

In contrast, changes in BMI-for-age *z*-score in the elder group of patients revealed a statistically significant improvement at F/U 1 with a mean difference of 0.41 (95% CI: 0.27–0.55, *n* = 24, *p* < 0.0001), which was sustained at F/U 2 (mean difference 0.51, 95% CI: 0.24–0.79, *n* = 24, *p* < 0.001) (Table 3; Figure 4B). With respect to weight-for-age *z*-scores, a mean difference of 0.34 at F/U1 was observed (95% CI: 0.22–0.46, *n* = 24, *p* < 0.0001) and sustained at F/U 2 (mean difference 0.43, 95% CI: 0.22–0.64, *n* = 24, *p* < 0.001) (Table 3; Figure 5B).

The mean of individual changes between the two age groups regarding BMI-for-age z-score did not show statistically significant differences at F/U 1 (*p* = 0.046) or F/U 2 (*p* = 0.082) after correction for multiple testing. With regard to weight-for-age z-scores the elder patients experienced a significantly greater increase at F/U 1, whereas the difference between the two age-groups was insignificant at F/U 2 (*p* = 0.0771).

Height-for-age*z*-scores did not undergo a consistent significant change in either of the groups (Table 3). Solely, the children 6–11 years had an increase in height-for-age z-score between F/U 1 and F/U 2 (*p* = 0.0061).

#### Genotype groups (F/F *versus* F/MF)

Reductions in sweat chloride concentrations to <60 mmol/L and <30 mmol/L after ETI were found to be more prevalent among children with the F/F genotype (100.0% and 36.0%, respectively) compared to children with F/MF genotypes (42.1% and 5.3%, respectively). This observation was reflected by the significantly greater decrease in mean sweat chloride concentration in the F/F group (−69.4 mmol/L; 95% CI, −74.3–−64.9) compared to the patients with the F/MF genotype (−45.9 mmol/L; 95% CI, −54.2–−37.7) (*p* < 0.0001). (Table 3; Figure 2C).

Regarding nutritional status, absolute changes in BMI-for-age and weight-for-age z-scores showed a marked separation of the two genotype groups in favor of the F/MF group (Figure 4C; Figure 5C). Whereas weight for age z-score improvement in the F/F group was only significant at F/U 1 (0.13, 95% CI, 0.06–0.21) but not at F/U 2 (*p* = 0.06), patients with F/MF genotypes had significantly improved weight-for-age z-scores at both follow-up visits (0.40 and 0.58 relative to baseline respectively, with *p* = 0.0001 and *p* = 0.0002).

F/F patients presented a BMI-for-age z-score improvement of 0.17 at F/U 1 (95% CI, 0.09–0.24, *p* < 0.0001), which was not sustained at F/U 2 (mean difference to baseline 0.12, *p* = 0.1113) (Figure 4C). On the contrary, patients with F/MF genotype presented a significant and sustained *z*-score improvement at F/U 1 and F/U 2 (mean difference 0.51 and 0.72, respectively, with *p* < 0.001 and *p* = 0.0003).

Again, height-for-age*z*-scores did not improve significantly for either of the genotype subgroups (Table 3).

Consistent with the statistically significant improvements in weight and BMI, changes in ppFEV_1_ compared to baseline were also greater among patients with F/MF genotype. At F/U 1 there was a trend toward a higher increase in ppFEV_1_ in patients with the F/MF genotype compared to the F/F group (15.5% *versus* 8.4%, *p* = 0.0397, n. s after correction for multiple testing). At F/U 2, ppFEV_1_ increased significantly by 17.3% in the F/MF group compared to 9.3% in the F/F patients (*p* = 0.0274). Both groups presented statistically relevant changes in ppFEV_1_ related to baseline at both follow-up examinations (*p* < 0.0001) (Figure 3C).

### Adverse events

ETI was generally well tolerated. A rash, considered attributable to ETI therapy, occurred in three patients, which was treated with antihistamines and did not lead to interruption of the modulator medication. At baseline, 11 patients had mild elevation of ALT or AST (<2xULN). On ultrasound before start of ETI, seven of the 11 patients with elevated hepatic enzymes had signs of CF liver disease and 2/11 patients were post liver transplantation. Of these eleven patients, only one with no morphologic signs of liver disease at baseline experienced a significant elevation of liver enzymes above 5xULN. ETI was stopped in this patient and successfully restarted with a reduced dose after normalization of liver enzymes. The other 10 patients did not have a further increase in liver enzymes. Eleven patients with normal liver enzymes at baseline experienced a mostly mild increase in ALT and/or AST. Interruption of ETI therapy was only required in two patients, both of whom were able to continue with a reduced dose. Of note, all patients with signs of liver cirrhosis on ultrasound at baseline tolerated ETI without a dose reduction.

Mild elevation of CK was a frequent finding and was less than 2-fold in all but one case. One 17-year-old boy had repetitive increases in CK up to 1026 U/L, often in association with physical exercise. Muscle MRI and CPT2-genotyping were normal and the boy continued with the regular dose.

One 16-year-old girl post liver transplantation experienced an increase in AP >6,000 U/L as well as diarrhea 3 weeks after the onset of ETI. Analysis of AP isoenzymes revealed mixed hepatic and bone origin of AP without intestinal AP. Liver enzymes, hepatic ultrasound and bone density were normal. ETI was interrupted and restarted after normalization of AP 6 weeks later without renewed elevation of AP in the further course. There were no other relevant changes in laboratory findings.

Another 16-year-old girl developed headaches and fatigue in association with the start of ETI. Ophthalmologic examination revealed bilateral papillary edema. Intracranial pressure was raised to 38 mmHg and was lowered to 20 mmHg during lumbar puncture. MRI of the brain showed no abnormalities. The vitamin A serum level was 374 μg/l (normal range 200—1,200). After 4 weeks, ETI was restarted in a reduced dose. To date, the girl has not reported a new onset of headaches or visual impairment. On follow-up fundoscopy, no papillary edema was detected. The same girl developed a psoriasis-like rash mainly on the trunk. Malassezia furfur was seen on microscopy, fungal culture was negative. Recently, skin biopsy revealed psoriasis vulgaris with no evidence of tinea corporis. The girl is scheduled for an appointment in the department of dermatology to discuss treatment options.

## Discussion

Treatment with ETI has been shown to be safe and effective in adolescents and adults with at least one *F508del* mutation (Nichols et al., 2022). ETI therapy is associated with a marked clinical benefit regarding pulmonary function, growth parameters, sweat chloride concentration (Sutharsan et al., 2022), nasal potential difference (Graeber et al., 2022a) and MRI parameters (Graeber et al., 2022b) compared to previously introduced CFTR modulators. Moreover, non-respiratory health-related parameters improved in adolescents and adults taking ETI (Fajac et al., 2022). Recently, two clinical trials documented the safety and efficacy of ETI in children ≥6 years with at least one *F508del* mutation (Zemanick et al., 2021; Mall et al., 2022). More recently, the results of the phase 3 open label trial including children aged 2–5 years taking ETI therapy were published (Goralski et al., 2023), demonstrating a significant improvement in LCI_25_ as well as reduced sweat chloride concentrations after 24 weeks of treatment. Outside clinical studies, compliance regarding the reliable drug intake is less closely monitored and as such more likely to be variable than under study conditions. Treatment efficacy may therefore differ from findings in controlled clinical trials. Real-life data in adolescents and adults (Nichols et al., 2022) have been presented including patients with severe lung disease in pre-approval compassionate use programs (Carnovale et al., 2022; Kos et al., 2022). To date, real life evidence of ETI therapy in children below 12 years of age is limited (Streibel et al., 2023).

### Pulmonary function tests

In this study, we report the intermediate-term efficacy and safety of ETI therapy in 46 pediatric CF patients in a CF clinic situated in the large metropolitan Rhine-Ruhr area. Under these post-approval conditions, the positive impact of ETI was comparable to that seen in the clinical trials with children (Zemanick et al., 2021; Mall et al., 2022) and adolescents (Middleton et al., 2019). In our patients, mean ppFEV_1_ was normal before start of ETI and improved by a mean of 11.4% across the study group. Zemanick et al. found an increase in ppFEV_1_ of 10.2% in children carrying at least one *F508del* mutation compared to baseline (Zemanick et al., 2021). In contrast to our study, these children had undergone a wash-out period of a previously prescribed modulator. In a placebo-controlled trial, Mall et al. reported a mean in-between-group difference of 11% ppFEV_1_ in children with a minimal function mutation compared to baseline (Mall et al., 2022). In adolescents who were homozygous for *F508del*, a mean increase in ppFEV_1_ of 13.8% was found (Middleton et al., 2019). Real world evidence given by Streibel et al. shows a mean increase in FEV_1_ z-score of 1.06 in children and adolescents after a mean of 4 months after start of ETI (Streibel et al., 2023). A significant association between pulmonary function improvements and structural MRI data in these patients was reported. Interestingly, in our study change in pulmonary function tests did not differ significantly between children 6–11 years compared to those ≥12 years. In contrast to this, children with the F/MF genotype experienced a more marked response to ETI regarding pulmonary function tests than those homozygous for *F508del* at both follow-up visits although this difference was only statistically significant at F/U 2 after correction for multiple testing. However, our results are in line with previous data (Nichols et al., 2022) and most probably reflect the modulator-naïve state prior to start of ETI in patients with the F/MF genotype. While average ppFEV_1_ in our patients was normal at baseline, we like others (Salvatore et al., 2022) found that the subgroup of children with impaired function experienced the largest increase in ppFEV_1_, again underlining the particular therapeutic value of ETI in these patients. We cannot reproduce the findings of Nichols et al. (Nichols et al., 2022), who described a significant correlation between the decrease in sweat chloride concentration and the increase in ppFEV_1_ after 6 months follow-up in adolescent and adult PwCF. It remains unclear, whether in our study this effect could have been demonstrated with a larger patient group.

In patients with normal pulmonary function, ventilation inhomogeneities are detected by means of the LCI. Regrettably, we are unable to report a consistent LCI data set in our study. In the literature, treatment with ETI was associated with a significant improvement in the LCI (Zemanick et al., 2021; Graeber et al., 2022b) in excess of that previously reported for LUM/IVA in young children (Ratjen et al., 2017). Recently, a significant LCI improvement was demonstrated in children aged 2–5 years after 6 months of ETI treatment (Goralski et al., 2023). Streibel et al. reported an improvement in LCI and MRT-derived ventilation and perfusion measures after a mean follow-up of 4 months after start of ETI therapy (Streibel et al., 2023).

### Growth parameters and exocrine pancreatic function

Improving and maintaining growth is of prognostic relevance regarding pulmonary function and survival in children with CF (Konstan et al., 2003; Vieni et al., 2013). In our study, mean z-scores for weight and BMI were normal at baseline, partially reflecting the effect of LUM/IVA (Hoppe et al., 2021) before start of ETI, and increased significantly after three and 6 months. In line with Zemanick et al., mean height z-scores remained unchanged after three to 6 months (Zemanick et al., 2021). The two age groups showed comparable effects of ETI on BMI and weight. Patients with the F/MF genotype experienced a greater increase in weight for age-z-score and BMI for age-z-score than those homozygous for *F508del*, which - as in the case of ppFEV_1_ - points to the modulator-naïve state of this group. The beneficial effects on the nutritional status in our patients exceeded those seen with LUM/IVA in children with the F/F genotype (Hoppe et al., 2021).

To date, we are not aware of any patient in our study reaching pancreatic sufficiency. However, fecal elastase was not systematically assessed in our patients. In the literature, there are reports of restored pancreatic function when using LUM/IVA (Vieni et al., 2013), in school-aged children taking IVA (Nichols et al., 2020) and even more so when IVA was introduced from the age of 4 months (Davies et al., 2021). Consequently, CFTR modulator therapy should be started as early as possible to maintain exocrine pancreatic function (Dave et al., 2021). So far, ETI does not seem to restore exocrine pancreatic function in children ≥12 years of age (Schwarzenberg et al., 2022). Data on fecal elastase were not reported in the pediatric trials (Mall et al., 2022; Nichols et al., 2022) including children ≥6 years of age. In children aged 2–5 years the mean increase in fecal elastase was 39.5 μg/g, with 6/75 children having a fecal elastase >200 μg/g after 6 months of ETI therapy (Goralski et al., 2023).

### Sweat chloride

The sweat chloride concentration, a surrogate measure for the CFTR function (Elborn, 2016), is an important outcome parameter when assessing the efficacy of modulator therapy. With IVA, sweat chloride was reduced below the threshold for the diagnosis of CF (Ramsey et al., 2011). The modulators LUM/IVA and TEZ/IVA resulted in less impressive reductions in sweat chloride but offered a modulator option for a large group of patients carrying two *F508del* mutations or a residual function mutation. With the advent of ETI therapy, the reduction in sweat chloride was comparable to that seen with IVA (Heijerman et al., 2019; Middleton et al., 2019). In children aged 6–11 years treated with ETI, a mean change in sweat chloride of 61 mmol/L was seen with an even greater mean reduction of 70 mmol/l in *F508del* homozygous patients (Zemanick et al., 2021). In our study, mean sweat chloride concentration decreased by 59 mmol/l across all patients, with a more pronounced in effect in those carrying the F/F genotype. There was no relevant difference in sweat chloride reduction between the age groups. In four of the 12 patients with a sweat chloride concentration >60 mmol/l during ETI treatment, suboptimal compliance in several fields had been a recognized problem. Consequently, dietary advice was repeated regarding the intake of ETI and a repeat sweat test was scheduled.

### Adverse events

Analysis of the adverse effects associated with ETI revealed a good safety profile as previously described for children (Zemanick et al., 2021; Goralski et al., 2023), adolescents and adults (Heijerman et al., 2019; Middleton et al., 2019). Adverse events thought to be related to ETI therapy were three transitory rash incidents in children without any other sign of acute illness. The children and their parents opted to continue the modulator medication without interruption or dose reduction. The use of an oral antihistaminic agent was considered beneficial. While rash events are known to occur frequently when initiating ETI therapy (Heijerman et al., 2019; Middleton et al., 2019) even when LUM/IVA or TEZ/IVA had been tolerated without rash, other dermatologic problems such as acne have only recently been reported in the context of ETI therapy (Hudson et al., 2022). The causal relation remains to be clarified. Regarding the new onset of psoriasis seen in our patient thus far there is no published evidence to support a connection to ETI therapy.

### Elevation of liver enzymes and creatinkinase

CFTR modulator therapy containing ivacaftor is well recognized to affect hepatic enzymes. Only a small percentage of patients cannot tolerate the full ETI dose due to consistently elevated AST or ALT. All CFTR modulators are metabolized by the CYP3A4 und CYP3A5 pathway, for which extensive pharmacogenetic heterogeneity exists. At this time, there is scarce information on drug plasma levels and their potential role in explaining side effects (Choong et al., 2022). In our study, only three patients needed to reduce the ETI dose because of hepatic toxicity. Pharmacogenetic testing revealed that two of these children had a reduced CYP3A4 activity while one boy with repeated elevation of liver enzymes had increased CYP3A4 und CYP3A5 activity. A recent study observed no connection between increased modulator plasma levels and reduced CYP 3A activity (Guimbellot et al., 2022). Three of our adolescent patients received ETI post liver transplantation. In this patient group, careful monitoring is necessary in order to diagnose pharmacologic interactions with immunosuppressive agents. No guidelines exist for the use of ETI in patients post liver transplant. Recently, a case series reported good tolerability of ETI therapy in post LTX patients with varying dose regimes and good clinical benefit (Ragan et al., 2022). In our limited experience, full dose ETI was tolerated without noticeable toxicity and immunosuppressive therapy remained well controlled. Elevation of CK is another well-known side effect of CFTR modulator therapy (Heijerman et al., 2019; Middleton et al., 2019; Zemanick et al., 2021) and is often asymptomatic. In one of our patients, CK was elevated more than usually seen but no predisposing condition was identified in the boy and therapy was continued without detrimental health effects.

### Pseudotumor cerebri

We report the case of a 15-year-old normal-weight girl with new onset of pseudotumor cerebri, also referred to as idiopathic intracranial hypertension (IIH) (Sheldon et al., 2017). Reports from the pre-modulator era (Obeid et al., 2011) of CF children with IIH were limited to cases with severe malnutrition and Vitamin A deficiency. To the best of our knowledge, there have been four reports of one adult and three adolescents developing intracranial hypertension while taking ETI (Miller and Foroozan, 2022; Wisniewski et al., 2022). Three patients displayed variable degrees of Vitamin A serum level elevations while one patient died due to complications of a previously unknown intracranial malformation. In our patient, the vitamin A serum levels were normal before and after onset of ETI, and no other substances linked to the development of IIH, e.g., spironolactone or tetracyclines had been prescribed (Sheldon et al., 2017). At this time, the onset of IIH in our patient remains unexplained.

### Limitations

We acknowledge several limitations of the present study. First, the data presented were collected retrospectively in a single center explaining the small number of patients compared to the multicenter clinical trials. Since all eligible children with CF were advised to start ETI therapy, data from a control group are not available. Due to the retrospective nature of this work, some relevant measurements such as fecal elastase or LCI could not be included in the analysis. Moreover, the follow-op duration of 6 months was too short to draw conclusions on the long-term term effect of ETI therapy in our patients. Data presented were partly collected during the COVID-19 pandemic, and contact restrictions may have reduced pulmonary exacerbation rates in our patients as previously described (Patel et al., 2021). However, this outcome is not reported here. Since our clinical surveillance routine was not significantly modified during the pandemic we do not assume that our data were noticeably affected during this period.

### Outlook

Treatment with ETI has opened a new perspective to children with CF and one *F508del* mutation. In view of the potentially life-long therapy, further prospective investigations are required to assess the long-term effects of ETI on the mental and physical health of growing children. Since safety and efficacy of ETI were recently reported in children two to 5 years of age ETI therapy should soon be offered to this age group to prevent the onset of chronic organ damage. The treatment of children with non-*F508del* mutations continues to pose a great challenge emphasizing the importance of identifying non-*F508del* genotypes responsive to ETI or future CFTR modulators.

## Data Availability

The raw data supporting the conclusions of this article will be made available by the authors, without undue reservation.

## Abbreviations

BMI, body mass index; CF, cystic fibrosis; ETI, elexacaftor, tezacaftor, ivacaftor; CI, confidence interval; F/F, Patients homozygous for F508del; F/MF, Patients heterozygous for F508del and a minimal function mutation; F/U 1, Follow-up visit after 3 months of ETI treatment; F/U 2, Follow-up visit after 6 months of ETI treatment; IVA, ivacaftor; LUM, lumacaftor; ppFEV1, percent predicted FEV1; N.s, not statistically significant; SD, standard deviation; TEZ, tezacaftor; ULN, upper limit of normal.
